# Effects of Antisense Oligonucleotides against C-Reactive Protein on the Development of Atherosclerosis in WHHL Rabbits

**DOI:** 10.1155/2014/979132

**Published:** 2014-04-27

**Authors:** Qi Yu, Zhengcao Liu, Ahmed Bilal Waqar, Bo Ning, Xianghong Yang, Masashi Shiomi, Mark J. Graham, Rosanne M. Crooke, Enqi Liu, Sijun Dong, Jianglin Fan

**Affiliations:** ^1^Department of Molecular Pathology, Interdisciplinary Graduate School of Medicine and Engineering, University of Yamanashi, Yamanashi, Chuo-City 409-3898, Japan; ^2^Department of Histology and Embryology, Xi'an Medical University, Xi'an 710021, China; ^3^Department of Pathology, Shengjing Hospital of China Medical University, Shenyang 110003, China; ^4^Experimental Animal Center and Animal Models for Cardiovascular Diseases, Kobe University School of Medicine, Kobe 6500017, Japan; ^5^Isis Pharmaceuticals Inc., Carlsbad, CA 92008, USA; ^6^Research Institute of Atherosclerotic Disease and Laboratory Animal Center, Xi'an Jiaotong University School of Medicine, Xi'an 710061, China; ^7^Key Lab of Urban Environment and Health, Institute of Urban Environment, Chinese Academy of Sciences, Xiamen 361021, China

## Abstract

Increased plasma levels of C-reactive protein (CRP) are closely associated with cardiovascular diseases, but whether CRP is directly involved in the pathogenesis of atherosclerosis is still under debate. Many controversial and contradictory results using transgenic mice and rabbits have been published but it is also unclear whether CRP lowering can be used for the treatment of atherosclerosis. In the current study, we examined the effects of the rabbit CRP antisense oligonucleotides (ASO) on the development of atherosclerosis in WHHL rabbits. CRP ASO treatment led to a significant reduction of plasma CRP levels; however, both aortic and coronary atherosclerotic lesions were not significantly changed compared to those of control WHHL rabbits. These results suggest that inhibition of plasma CRP does not affect the development of atherosclerosis in WHHL rabbits.

## 1. Introduction

C-reactive protein (CRP) is a classical plasma protein marker that is markedly elevated in the acute phase of inflammation, infection, and tissue damage and thus has been broadly used for monitoring and differential diagnosis [1, 2]. The major functions of CRP include its ability to bind to various ligands exposed to damaged tissue or bacteria (opsonization) for the enhancement of phagocytosis and activation of the complement pathway, thereby enabling it to exert both anti- and proinflammatory functions [2, 3]. CRP is mainly expressed by hepatocytes, and its synthesis is regulated at the posttranscriptional level by cytokines [4]. Ample data from both clinical and experimental studies have shown that a high level of plasma CRP is a risk factor as well as marker for cardiovascular diseases [5–9], although some studies failed to prove the risk of CRP compared to other risk factors. The JUPITER trial (Justification for the Use of Statins in Primary Prevention: an Intervention Trial Evaluating Rosuvastatin) showed that a lipid-lowering drug, rosuvastatin (Crestor), can significantly reduce the incidence of major cardiovascular events, even in apparently healthy subjects not exhibiting established risk factors such as hyperlipidemia, but with elevated high-sensitive CRP levels [10]. Regardless of this controversy, emerging evidence indicates that high levels of CRP may be potentially atherogenic [11, 12]. However, this hypothesis is under debate. Studies of transgenic mice (expressing either human or rabbit CRP) along with human CRP transgenic rabbits and CRP-deficient mice failed to provide a clear conclusion regarding whether CRP is atherogenic [13–23]. The major concerns about these animal studies are as follows: (1) mouse endogenous CRP is not physiologically active* in vivo* and (2) transgenic proteins are exogenous to animals, which may complicate the evaluation of CRP pathophysiological functions in these models [23]. In our previous study, we found that WHHL rabbits are an excellent model for the study of CRP and its relationship with atherosclerosis because they have higher levels of plasma CRP and immunoreactive CRP proteins are present in lesions of atherosclerosis [24]. In addition, rabbit CRP has 74% homology with human CRP [1] and rabbit CRP levels are highly inducible and responsive during the inflammatory reaction [25]. To examine whether CRP is involved in the development of atherosclerosis and whether therapeutic strategies to lower CRP levels are useful for treating atherosclerosis, we intravenously injected the rabbit CRP antisense oligonucleotides (ASOs) into WHHL rabbits. Using two different-aged WHHL models, we examined (1) whether CRP ASOs could reduce the plasma levels of CRP and (2) whether CRP lowering would affect the initiation and progression of aortic atherosclerosis and coronary atherosclerosis. However, we did not identity antiatherogenic effects of CRP antisense, suggesting that CRP is not an atherogenic factor or a therapeutic target for the treatment of atherosclerosis.

## 2. Materials and Methods

Watanabe heritable hyperlipidemic (WHHL) rabbits [26] were bred in a closed colony at Kobe University and housed in the animal facility of University of Yamanashi with a 12 h light/dark cycle at 23°C and 55% humidity. They were fed with a standard chow diet (CR-3), containing 17.6% protein, 4.1% fat derived from soybean oil, and 10.1% fiber (CLEA Japan, Inc., Tokyo, Japan) and had free access to water. All animal experiments were performed with the approval of the Animal Care Committee of the University of Yamanashi and conformed to the Guide for the Care and Use of Laboratory Animals published by the US National Institutes of Health. Rabbit CRP antisense oligonucleotides (ASO, 5′ATAAGCAAGCAAACACCC3′, no. 280290) and mismatched control oligonucleotides (5′CCTTCCCTGAAGGTTCCTCC3′, no. 141923) were designed and synthesized by ISIS Pharmaceuticals Inc. (Carlsbad, CA) [27]. ASO 280290 was selected among 100 candidate oligonucleotides and doses aimed at obtaining maximally inhibitory efficacy were screened using cultured rabbit hepatocytes. For* in vivo* studies, CRP ASOs were dissolved in saline solution and intravenously injected into WHHL rabbits through ear veins (60 mg/Kg BW/week) twice a week for 16 weeks. Control mismatched oligonucleotides were injected in the same way as CRP ASOs.

## 3. Experimental Design and Analysis

To examine whether rabbit CRP ASO administration could affect the development of atherosclerosis, we designed and performed two experiments. For the first experiment, we used young WHHL rabbits aged 3~4 months (*n* = 9 for each group, containing 5 males and 4 females). These WHHL rabbits started to develop aortic atherosclerosis, which represents the so-called early-stage lesions, such as fatty streaks in humans [28]. Therefore, we could examine whether CRP ASOs had any effects on the initiation and prevention of atherosclerosis. For the second experiment, relatively old WHHL rabbits (8–11 months old, *n* = 10 for each group) were used. These old WHHL rabbits developed advanced lesions, which are similar to human atherosclerotic plaques (including lipid cores, fibrous caps, and shoulders) [28]. In this way, we could clarify whether CRP ASO had any influence on the progression of the advanced lesions of atherosclerosis.

Plasma total cholesterol (TC), triglycerides (TG), and HDL-cholesterol (HDL-C) were determined using Wako assay kits (Wako Pure Chemical Industries, Osaka, Japan). For the analysis of lipoprotein profiles, plasma was resolved by electrophoresis in 1% agarose universal gels (Helena Laboratories, Saitama, Japan), and then the gels were stained with Fat Red 7B [29]. Plasma lipoproteins were also analyzed using high-performance liquid chromatography (HPLC) by Skylight Biotech, Inc. (Tokyo, Japan), at the end of the experiment. Plasma CRP levels were quantified using CRP enzyme-linked immunosorbent assay (ELISA) kits (Shibayagi Co., Ltd., Gunma, Japan) [30]. Liver mRNA expression levels of CRP were analyzed by real-time reverse transcriptase-polymerase chain reaction (RT-PCR), as described previously [30]. All rabbits were sacrificed after 16 weeks and their aortas and hearts were dissected for examination of atherosclerotic lesions using the standard method established in our laboratory [23, 31].

### 3.1. Statistical Analysis

All values are expressed as the mean ± SD and statistical significance was determined using Student's* t*-test. In all cases, statistical significance was set at *P* < 0.05.

## 4. Results

### 4.1. CRP ASO Effects on* Young* WHHL Rabbits

**Figure 1:**
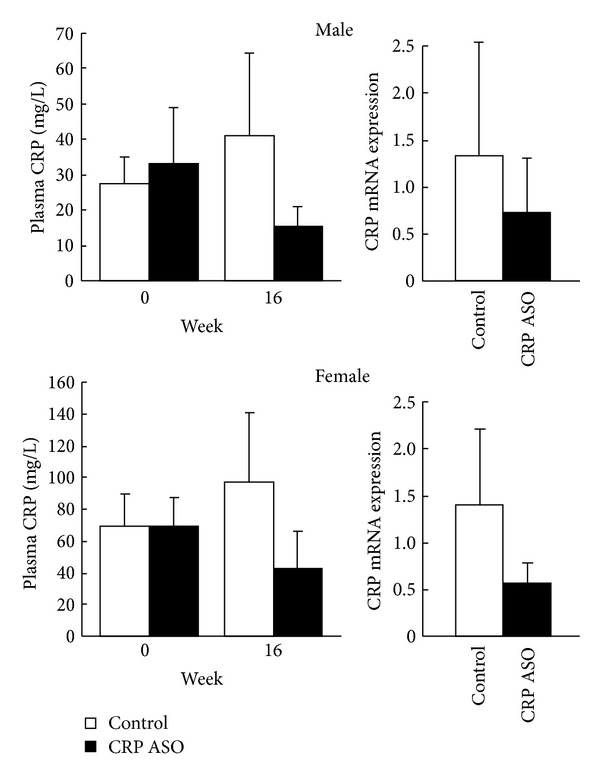
Plasma levels of CRP in WHHL rabbits and real-time RT-PCR analysis of hepatic mRNA of CRP expression. Plasma CRP levels were measured before and after treatment with CRP ASOs for 16 weeks. CRP mRNA expression in the liver was quantified at 16 weeks. The values are expressed as the mean ± SD. *n* = 4-5 for each group.

We first measured the plasma levels of CRP after CRP ASO treatment. As reported in our previous study, WHHL rabbits at 4 months exhibited higher plasma levels of CRP than JW wild-type rabbits [30]. As shown in Figure 1 (left panels), WHHL rabbits had about 10-fold (in males) and 20-fold higher CRP (in females) levels at 4 months than JW rabbits (3.15 ± 0.8 mg/L) [30]. 16 weeks later, plasma levels of CRP were further increased by 143% (on average in males and females), suggesting that these increased CRP levels of WHHL rabbits were accompanied by (or correlated with) the development of aortic lesions induced by hypercholesterolemia. Treatment with CRP ASOs for 16 weeks led to the reduction of plasma CRP by 61% in males and 56% in females compared with that of the controls, although the differences were not significant (*P* = 0.06). Reduced plasma levels of CRP were consistent with lower hepatic expression of CRP mRNA, as shown by RT-PCR analysis (Figure 1, right panels).

**Figure 2:**
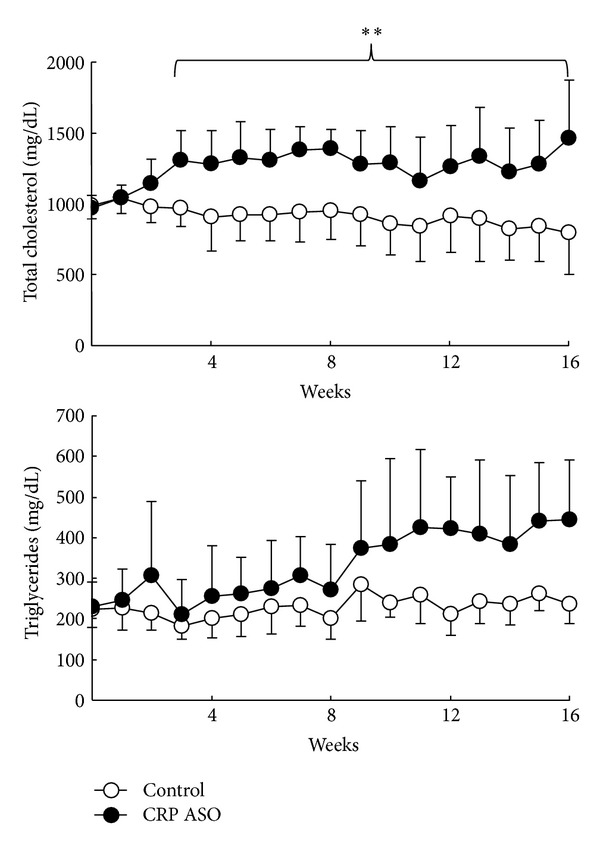
Analysis of plasma levels of lipids. CRP ASO treatment increased plasma lipids (TC and TG). Data are combined from male and female rabbits and expressed as the mean ± SD. *n* = 4-5 for each group.

**Figure 3:**
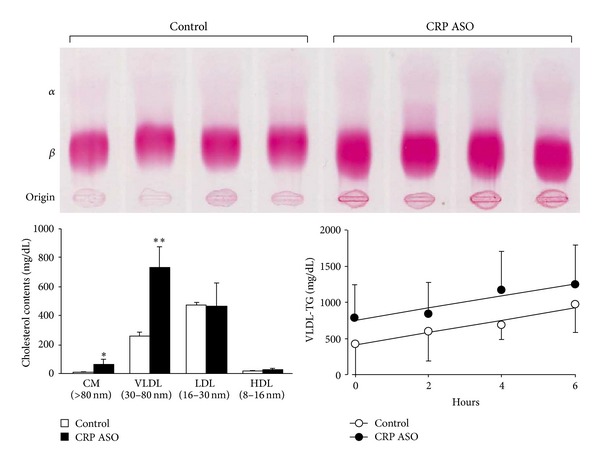
Analysis of lipoprotein profiles. Lipoprotein profiles were analyzed by agarose gel electrophoresis (top) and HPLC (bottom left). Post-Triton VLDL production rate in fasting rabbits was measured. Blood was drawn at 0 minutes (before administration of Triton WR-1339) and 2, 4, and 6 hours after Triton WR-1339 injection. VLDL-TG (*d* < 1.006 g/mL) contents were quantified (bottom right). ASO-treated rabbits showed increased levels of *β*-migrating particles (both VLDL and chylomicron remnants) at the origin. VLDL synthesis rate (expressed by the slope of each line) was the same in ASO-treated rabbits as in the control. Data are expressed as the mean ± SD * or **P < 0.05 or 0.01 versus control. *n* = 4-5 for each group.

In spite of this, we found that CRP ASO-treated WHHL rabbits had somewhat higher plasma lipids including TC (starting from 3 weeks, *P* < 0.05) and TG (starting from 10 weeks, *P* < 0.05) than controls, (Figure 2) while HDL-C levels were unchanged (data not shown). Analysis of lipoprotein profiles revealed that increased plasma lipids in CRP ASO-treated WHHL rabbits were mainly caused by significantly increased very low density lipoproteins (VLDLs) (*P* < 0.01) and chylomicron remnant contents (*P* < 0.05) (Figure 3). To elucidate the possible mechanisms, we measured the rates of VLDL secretion in fasting animals* in vivo* using Triton WR-1339 to block hydrolysis of TG-rich lipoproteins by lipoprotein lipase [32]. The base line levels of VLDL-TG of ASO-treated WHHL rabbits were higher than those of controls; however, VLDL synthesis rate afterwards was similar to that in controls (Figure 3).

**Figure 4:**
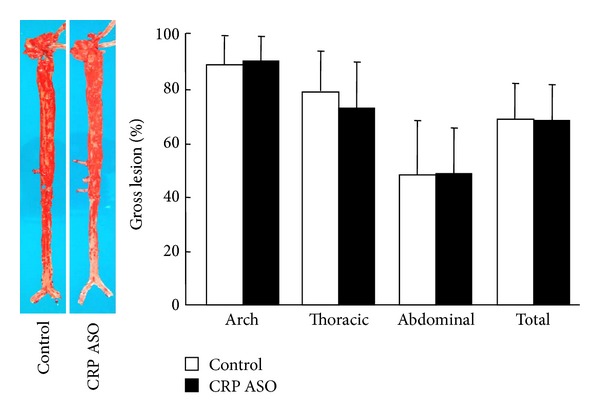
Analysis of aortic lesions. Representative photographs of pinned-out aortic trees stained with Sudan IV from CRP ASO-treated and control rabbits are shown (left), and aortic atherosclerotic lesions (defined by sudanophilic area) on the surface were quantified with an image analysis system (right). *n* = 9 for each group.

**Figure 5:**
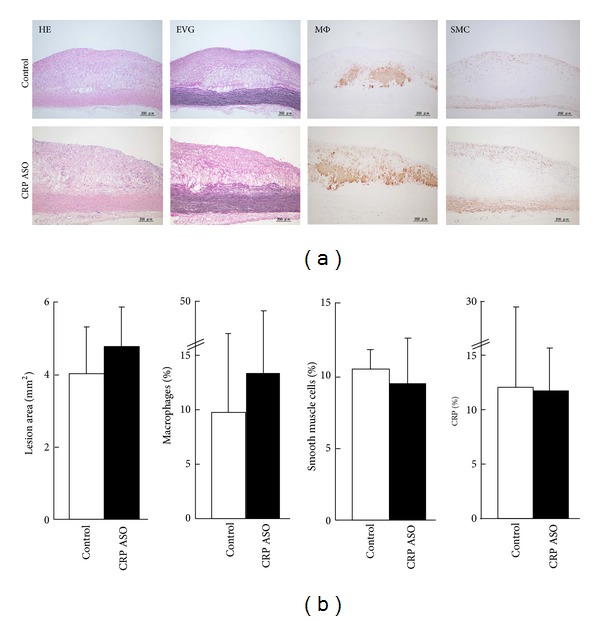
Microscopic analysis of the aortic lesions. Representative micrographs of the aortic lesions from CRP ASO-treated and control rabbits (a). Serial paraffin sections were stained with hematoxylin-eosin (HE) and elastica van Gieson (EVG) or immunohistochemically stained with monoclonal antibodies (mAbs) against either macrophages (M*φ*) or *α*-smooth muscle actin for smooth muscle cells (SMC) or rabbit CRP. Intimal lesions on EVG-stained sections and positively immunostained areas of macrophages; SMC and CRP were quantified with an image analysis system (b). *n* = 9 for each group.

After rabbits were sacrificed, we compared the aortic lesions and examined the histological features under a light microscope. As shown in Figure 4, ASO treatment did not change the aortic* en face* lesion areas in all parts compared to those of controls. We further compared the microscopic lesion size and histological features. Quantitative analysis of the microscopic lesions and macrophage and smooth muscle cell contents along with CRP immunoreactive protein deposition did not reveal any significant differences between CRP ASO-treated WHHL rabbits and controls (Figure 5).

### 4.2. CRP ASO Effects on* Old* WHHl Rabbits

**Figure 6:**
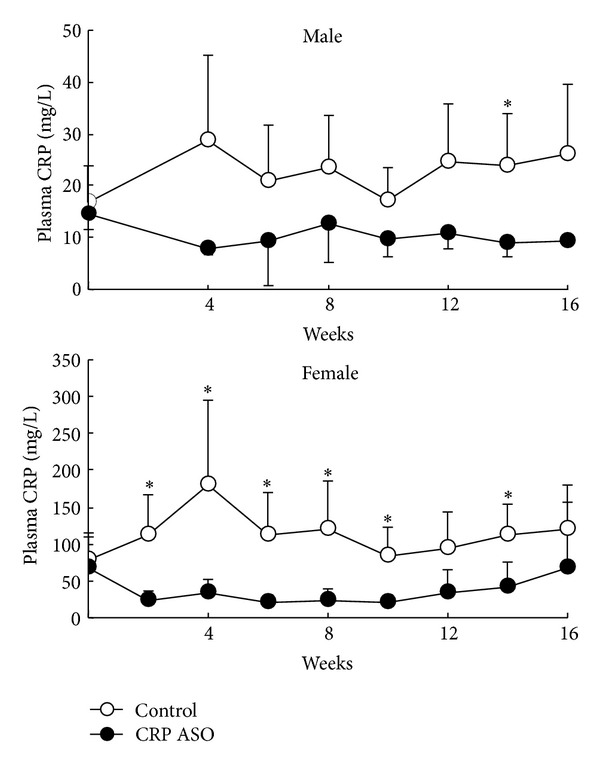
Plasma levels of CRP in WHHL rabbits. Plasma CRP levels were measured every two weeks after CRP ASO treatment. The values are expressed as the mean ± SD. *n* = 5 for each group. *P < 0.05 versus control.

In the second experiment, we treated WHHL rabbits aged 8~11 months with CRP ASOs. These old rabbits showed extensive atherosclerotic lesions in both aortas and coronary arteries [28] accompanied by high levels of CRP [30]. After treatment with CRP ASOs for 16 weeks, plasma CRP levels were consistently lower than in controls (Figure 6).

**Figure 7:**
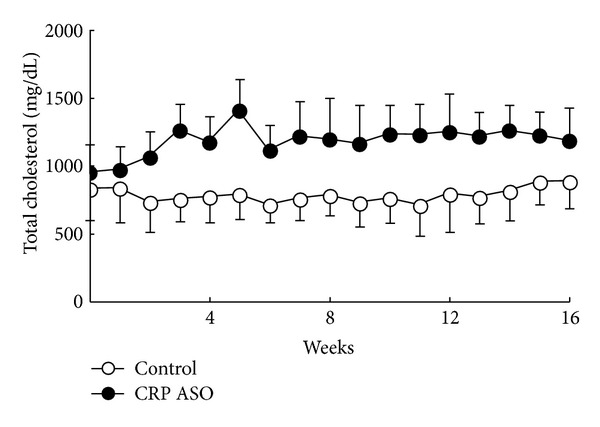
Plasma levels of total cholesterol. Data are combined from male and female rabbits and expressed as the mean ± SD. *n* = 10 for each group.

**Figure 8:**
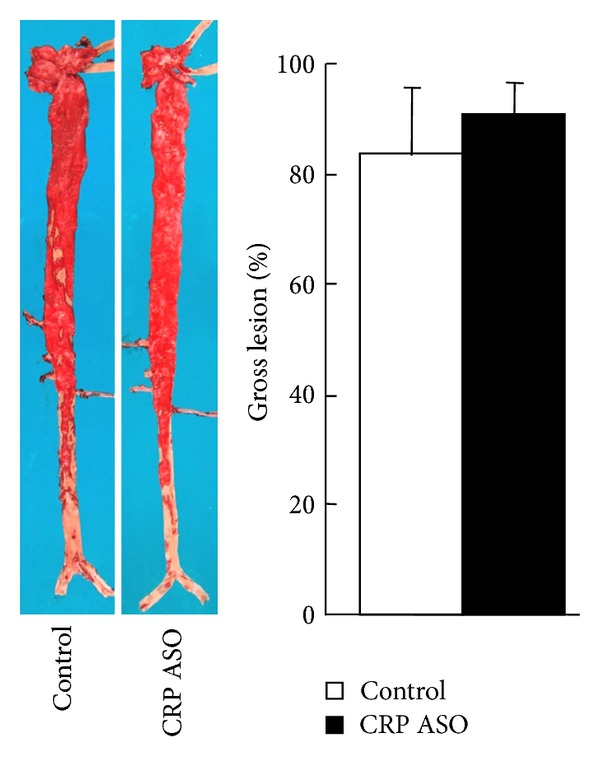
Analysis of aortic atherosclerosis. Representative photographs of pinned-out aortic trees stained with Sudan IV from CRP ASO-treated and control rabbits are shown (left), and aortic atherosclerotic lesions (defined by sudanophilic area) on the surface were quantified with an image analysis system (right). Data are combined from male and female rabbits and expressed as the mean ± SD. *n* = 10 for each group.

**Figure 9:**
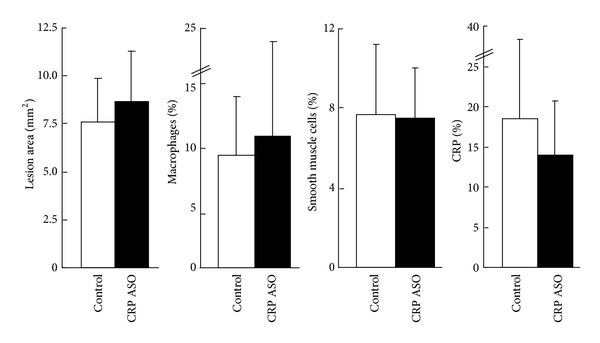
Microscopic analysis of the aortic lesions. Serial paraffin sections were stained with hematoxylin-eosin (HE) and elastica van Gieson (EVG) or immunohistochemically stained with monoclonal antibodies (mAbs) against either macrophages (M*φ*) or *α*-smooth muscle actin for smooth muscle cells (SMC) or rabbit CRP. Intimal lesions on EVG-stained sections and positively immunostained areas of macrophages; SMC and CRP were quantified with an image analysis system. Data are combined from male and female rabbits and expressed as the mean ± SD. *n* = 10 for each group.

**Figure 10:**
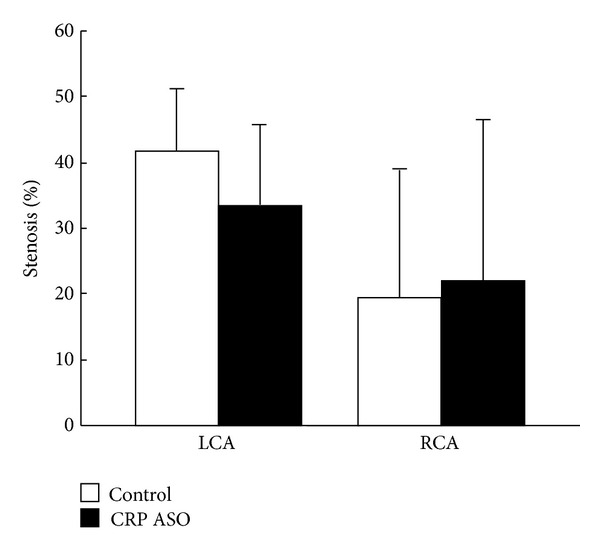
Analysis of coronary atherosclerosis. The heart was cut into 7 blocks, and blocks I and II containing left and right coronary trunks were sectioned in 500 *μ*m intervals (3 sections from each block) and stained with EVG. Coronary stenosis (lesion area/total lumen area × 100(%) was measured and is expressed as percentage. LCA indicates left coronary artery trunks; and RCA, right coronary artery trunks.

Similar to the first experiment, plasma levels of TC were higher in the CRP ASO-treated group than that in controls (Figure 7), while TG and HDL-C levels were unchanged (data not shown). Regardless of prominently lower CRP levels, we did not find any differences between ASO-treated and control groups in both aortic gross and microscopic lesion areas (Figures 8 and 9). Histological examination revealed that CRP immunoreactive protein contents were slightly reduced in the lesions of CRP ASO-treated WHHL rabbits, but without statistical significance (Figure 9). Because old WHHL rabbits developed coronary atherosclerotic lesions, we further compared the coronary lesions (expressed as stenosis percentage) and found that left coronary stenosis was slightly less in CRP ASO-treated WHHL rabbits, although the difference was not statistically significant (Figure 10).

## 5. Discussion

C-reactive protein (CRP) is not only a predictor but also a potential risk factor of cardiovascular events [33]. Several lines of evidence showed that CRP may modulate the vascular functions and thereby influence the initiation and progression of atherosclerosis [11, 34]. On the other hand, many controversial and contradictory results from both human and experimental animals have been published on the effects of CRP on atherosclerosis [18, 23, 35]. In the current study, we first developed rabbit CRP antisense oligonucleotides and then evaluated their effects on WHHL rabbits, a well-established model for the investigation of atherosclerosis. Although CRP ASOs could reduce the plasma levels of CRP through inhibiting hepatic CRP synthesis, we failed to demonstrate any beneficial (antiatherogenic) effects caused by CRP lowering: CRP ASO treatment did not change the aortic and coronary atherosclerosis in two groups of WHHL rabbits compared with that of controls. In spite of this, CRP ASO did not affect the lesion cellular components as well. Therefore, these results are consistent with our previous study using cholesterol-fed human CRP transgenic rabbits [23] and further strengthen the notion that CRP is not an atherogenic factor but rather an inflammatory marker [36]. It is also unlikely that CRP can be a therapeutic target for the treatment of atherosclerosis. These observations are consistent with the current clinical trial results showing that CRP inhibitors can reduce plasma CRP levels by ~80% in normal subjects, as well as, endotoxin challenged and atrial fibrillation patients while other key cytokines, signs, and symptoms remained entirely unchanged in the endotoxin challenged subjects (Graham, M. personal communications).

It should be pointed out, however, that the current results cannot rule out the possibility that CRP may be involved in other inflammatory diseases.

Unexpectedly, we found that CRP ASO treatment elevated plasma lipids in WHHL rabbits due to enhancement of apoB-containing particle production. CRP ASO-induced lipid raising effect was not found in human clinical trials using CRP ASOs. It is currently unknown whether elevated plasma lipids are caused by CRP inhibition or CRP ASOs themselves (such as off-targeting effects).

In conclusion, we found that CRP lowering does not have significant influence on the initiation and progression of atherosclerosis in WHHL rabbits; thus, CRP may not be a therapeutic target for the treatment of atherosclerosis.
